# Facilitating trial recruitment: A qualitative study of patient and staff experiences of an orthopaedic trauma trial

**DOI:** 10.1186/s13063-019-3597-8

**Published:** 2019-08-09

**Authors:** Emma Elizabeth Phelps, Elizabeth Tutton, Xavier Griffin, Janis Baird, On behalf of the TrAFFix study co-applicants, Matthew L. Costa, Nicholas Parsons, Melina Dritsaki, May Ee Png, Juul Achten, Robin Lerner, Alwin McGibbon

**Affiliations:** 1 NDORMS, Kadoorie Centre, level 3, John Radcliffe Hospital, University of Oxford, Oxford, OX3 9DU UK; 2 Oxford University Hospitals NHS Foundation Trust, Oxford, UK; 3 Warwick Research in Nursing, University of Warwick, Coventry, UK; 4 MRC Lifecourse Epidemiology Unit, University of Southampton, Southampton, SO16 6YD UK

**Keywords:** Qualitative, Interviews, Experience, Recruitment, Trials

## Abstract

**Background:**

Qualitative research has been used to explore patients’ and healthcare professionals’ experiences of surgical randomised controlled trials (RCTs). From this research, reasons why patients accept or decline participation and barriers to engaging clinicians in trials have been identified. In a trauma setting, recruitment to surgical trials can be particularly difficult as patients may require urgent treatment and their ability to consider their options, ask questions and reach a decision may be hindered by the impact of their injury. Little research, however, has explored patients’ and healthcare professionals’ experiences of surgical RCTs in a trauma setting. This study aimed to understand patients’ and staff’s experiences of an orthopaedic trauma trial.

**Methods:**

We carried out semi-structured interviews with 11 patients and 24 staff (10 surgeons and 14 research associates) participating in a UK multi-centre feasibility trial comparing intramedullary nails versus distal locking plates for fractures of the distal femur (TrAFFix). Interviews explored patients’ experience of TrAFFix and their reason for participating and staffs' experience of recruiting to TrAFFix and trauma trials more generally. Interviews were audio recorded and transcribed verbatim. Transcripts were analysed using thematic analysis.

**Results:**

Three themes were identified. These were i) navigating research with patients after orthopaedic trauma, ii) knowing that it is the right decision and iii) making it work. These themes reflect: i) how research associates supported and guided patients through the consent process enabling them to participate, ii) the difficulty in engaging surgeons in a trial when individual equipoise and experience of the interventions are low despite the presence of community equipoise and iii) the way in which research teams worked together and encouraged the development of a research culture within the clinical teams in order to facilitate recruitment.

**Conclusions:**

Our findings highlight the pivotal role of research associates (RAs) in facilitating trial recruitment. RAs supported patients to enable them to make a decision about participation and assisted in developing a research culture within the team by promoting studies and communicating research to clinical staff. Our findings also reinforce surgeons’ difficulty with equipoise and suggest that accepting community equipoise could facilitate recruitment.

## Background

Randomised controlled trials (RCTs) are widely accepted as the best method of evaluating the effectiveness of medical treatments [1]. However, recruitment to RCTs can be slower and more difficult than expected [2]. Qualitative research with patients and healthcare professionals has highlighted important barriers and facilitators to trial recruitment.

Barriers to recruitment include patients’ struggle to understand or accept randomisation, wishing to choose their preferred treatment or for their clinician to determine the best treatment for them [3–6].Clinicians’ willingness to include patients in a trial may also be undermined by several factors including: i) difficulty communicating trial aims and concepts, ii) concern about the implications of recruitment for the doctor patient relationship, iii) a lack of equipoise and iv) an increased work load [7].

Facilitators to trial recruitment include raising the study with patients early on, having a second clinician present, having an extra consultation to allow patients time for reflection, avoiding words relating to gambling or winners and losers and individualising information to the patient [5]. Furthermore, Horwood et al. [8] found research nurses provided valued support and could facilitate recruitment as they were a visual reminder of the trial, reduced burden on the clinical team and were available to provide information and clarify trial procedures.

Trials of surgical interventions can encounter additional challenges [9–11]. Surgical culture has been argued to be one of several barriers to participation in RCTs. Surgeons may be uncomfortable with equipoise, which is in conflict with their usual practice of providing the right answer for every patient [11]. Standardisation of procedures is not always feasible as surgeons vary in expertise, ability and preference. In trials comparing a surgical intervention to a non-surgical intervention, patients can have strong treatment preferences, making recruitment challenging. Furthermore, surgery is often irreversible, with patients who are found to have received the least effective intervention unable to change their treatment at the end of the study [9–11].

In a trauma setting, recruitment to surgical trials can be particularly difficult as patients may require urgent treatment outside normal working hours and their ability to consider their options, ask questions and reach a decision may be hindered by the psychological impact of their injury. Huxley et al. [12] highlighted challenges of recruiting patients to a trial of two treatments for hip fractures. They found that although patients were willing to participate, nearly all experienced some lack of recall about the study, with some not recalling participating at all [12]. Surgeons also reported “possible” barriers including the need for the correct resources out of usual working hours, the requirement for expertise in both surgical interventions and a discomfit in telling patients that they did not know the best treatment for hip fracture [12].

Although potential barriers to conducting research in this setting have been identified, little research has considered patient and staff experiences of surgical trials in trauma. Understanding patient and staff experiences may provide insight into how trauma trials work in practice and how recruitment can be facilitated. Therefore, we explored patients’ experiences of participating and staff experiences of being involved in an orthopaedic trauma trial: TrAFFix [13].

## Methods

This study was part of a mixed methods process evaluation for the TrAFFix study [13]. TrAFFix was a multi-centre, randomised controlled feasibility trial of two surgical methods used to fix fractures of the distal femur (locking plate fixation and intramedullary nail fixation). The trial was registered with the International Standard Randomised Controlled Trials Number Registry (ISRCTN92089567). TrAFFix was open for recruitment in seven centres across England between October 2016 and July 2017. During this time, 25 of 85 eligible patients were invited to participate in TrAFFix (either prospectively before surgery or retrospectively after surgery, having been included with agreement from a personal or nominated consultee) and 23 were randomised. When patients lacked capacity a relative/friend (personal consultee) who knew the patient was informed about the study and asked if in their opinion the patient would object to taking part. If they felt they would not object, if able, and were willing to sign the personal consultee form, the patient was included in the study. If a personal consultee was not available, a nominated consultee, normally a surgical consultant who knew the patient but was not involved with the study, advised the team about the patient’s participation. If the patient regained capacity at any time, they provided written informed consent to continue in the study. As part of the process evaluation, we interviewed patients recruited to the study or their personal consultee and staff (namely surgeons and research associates (RAs) involved in the study). Personal consultees were included as we wished to understand, in the emergency situation, what it was like for them to advise the team about their relative’s/friend’s participation. TrAFFix and the embedded process evaluation were approved by The Wales Research Ethics Committee (REC reference 16/WA/0225). The findings from TrAFFix, including key points from the process evaluation, are published elsewhere [14]. This study reports the qualitative analysis of data collected from interviews with patients and staff involved in TrAFFix.

Interviews drew upon phenomenology [15], which seeks to understand individuals’ lived experience of a phenomenon of interest. We wanted to understand patients’ experience of participation in a surgical trial after a fracture and staff involvement in an orthopaedic trauma trial. For patient and consultee interviews, we intended to obtain a purposeful sample to obtain a range of ages, sex, both treatments, all study sites and those with a breadth of experience. However, within 2 months of the start of the study it was clear recruitment would be difficult (overall patients randomised *n* = 23) so all patients or consultees (none were excluded) who were invited to participate in TrAFFix were asked if they could be approached by a researcher about taking part in an interview. Patients and consultees were asked about taking part in an interview at the time of their consent discussion for the main trial. Those who agreed to be approached were sent an information sheet about the interview by post and were then contacted by the researcher by telephone to answer any questions and, if they wanted to take part, arrange a time for the interview. The intention was to interview patients/consultees in the first 6 weeks post-injury and again at 4 months to obtain an understanding of their participation in the study and early/later recovery. Three patients felt well enough to undertake both interviews. The remainder (*n* = 8) took part in an interview up to five months post injury due to the slow pace of recovery, prolonged hospitalisation or transition to care homes prior to returning home. Patients needed to feel well enough and have the energy to talk to the researcher; some delayed their interviews until they felt better. For staff interviews, we used a purposive sampling strategy inviting key staff from each centre involved in the study to participate. This took place towards the end of the study when they had experience of recruiting to the study. Interviews were semi-structured and used a brief topic guide, which was refined after the first few interviews. For patient interviews topics included their experience of i) injury, ii) recovery and iii) taking part in TrAFFix. Staff interviews explored experiences of trauma trials in general as well as staff experiences of TrAFFix. All staff were asked about their experience of i) recruiting to TrAFFix and other trauma trials, ii) their experience of explaining research studies to patients and iii) their views on their colleagues’ engagement with research and TrAFFix. Research associates were also asked about their experience of consenting patients who had a nominated consultee and their experience of follow-up questionnaires while surgeons were also asked about their experience of the two interventions, their views on rehabilitation and their views on blinding. Interviews varied in length from 20 to 70 min depending on the circumstances and were conducted face to face (hospital or home) or by telephone. Interviews were audio-recorded and transcribed verbatim, where participants agreed.

The data were managed using the software package Nvivo 10. Our approach to analysis was inductive, with themes derived from the data. Immersion in the data was achieved prior to coding by studying the transcripts. Initial codes were developed by grouping sentences and paragraphs of similar meaning together. Similar codes were then grouped together into categories and by comparing within and across the categories themes were developed. Data saturation was achieved in the categories and themes. An experienced, female qualitative researcher with a PhD and a background in psychology undertook data collection and analysis. Throughout analysis, four researchers from different disciplines, namely nursing, public health, psychology and surgery, met regularly to discuss the emerging themes. This allowed us to be confident that our interpretation of the data reflected the experience of our participants.

### Patient and public involvement

A patient and public involvement (PPI) representative was a co-applicant on the funding application and a member of the trial management committee. We consulted PPI representatives on the design of patient-facing documents and the interview prompts using the UK Musculoskeletal Trauma PPI group. A one-day workshop was held with six PPI representatives and four researchers to develop an outline of the challenges in the patient pathway and sensitise the team to areas of interest/concern that might be explored in the interviews. This meeting was transcribed verbatim and a summary sent to the attendees and the management team. Discussion of the findings with members of the UK Musculoskeletal Trauma PPI group suggests there is resonance with the findings.

## Results

Nine patients and two personal consultees from five of the seven centres participating in TrAFFix were interviewed. Of the patients or their consultee (*n* = 23) from the main study, six declined to be approached to take part in an interview as they felt it was too much of a burden, while 17 agreed to be approached. Subsequently one of the 17 patients died, one withdrew before being approached by the researcher, two declined when approached due to fatigue and two could not be contacted. Of the patients who were interviewed or whose consultee was interviewed on their behalf, two were male and nine were female. They were all of white ethnicity and aged between 54 and 93 years (mean age of participants, 76.3 years). One patient who had capacity and provided consent to take part in the trial was frail and unable to withstand an interview. As she still wished to take part her daughter went through the interview questions with her and gave the answers to the researcher in writing. The team felt this compromise was important to ensure the contributions of frail patients were included. The characteristics of this patient are included in Table 1. Prior to their fracture five patients were classified as frail (either mildly (*n* = 1), moderately (*n* = 1), severely (*n* = 2), or very severely (*n* = 1)) using the Rockwood frailty scale [16].

**Table 1:** Participant characteristics

Participant	Age (years)	Pre-fracture mobility	Time since surgery	Interviewed
Participant 1	60–69	Freely mobile without aids	2 months; 6.5 months	Patient
Participant 2	70–79	Some indoor mobility but never outside without help	10 days; 2 months	Patient
Participant 3	80–89	Some indoor mobility but never outside without help	7 days; 5 months	Patient
Participant 4	90–99	Mobile outdoors with one aid	2.5 months	Consultee
Participant 5	80–89	Mobile outdoors with one aid	3 months	Patient
Participant 6	50–59	Mobile outdoors with one aid	1.5 months	Patient
Participant 7	60–69	Mobile outdoors with one aid	4 months	Patient
Participant 8	60–69	Freely mobile without aids	5 months	Patient
Participant 9	Unknown	Mobile outdoors with one aid	4.5 months	Patient
Participant 10	80–89	Some indoor mobility but never outside without help	3 months	Patient with help from consultee
Participant 11	90–99	Mobile outdoors with one aid	5 months	Consultee

Twenty-four members of staff participated in an interview; all of the seven centres had at least two members of staff who were interviewed. In five sites additional members of staff were identified who had a range of experience of recruiting to TrAFFix. No staff members declined to participate. At each centre, this included the chief or principle investigator (PI); a surgeon responsible for the day-to-day running of the study at their site and a member of staff whose role was to recruit patients to the study. Of the 24 staff who were interviewed, ten were surgeons and the remaining 14 staff, hereafter referred to as research associates (RAs) to protect anonymity, included research nurses, an RA, a physiotherapist, a research manager and a trial coordinator.

### Facilitating trial recruitment

Three themes that facilitate trial recruitment were identified within the data: i) navigating research with patients after orthopaedic trauma, ii) knowing that it is the right decision and iii) making it work. The codes, categories and themes within facilitating trial recruitment are presented in Table 2.

**Table 2:** The codes, categories and themes within facilitating trial recruitment

Theme	Category	Code
Navigating research with patients after orthopaedic trauma	Making sense of the study	Recall
		Randomisation
		Confusion and therapeutic misconception
	Enabling participation	Assessing capacity
		Involving family
		Consultees
Knowing it’s the right decision	Interpreting the eligibility criteria	Believing few patients are eligible
		Excluding eligible patients
	Surgeon preference	Appropriateness of interventions
		Surgical skill and experience
		Collective preferences
	Community equipoise	Accepting community equipoise
		Difficulty with individual equipoise
Making it work	Juggling activities	Minimising the impact of research on clinical staff
		Helping patients
	Balancing their own beliefs with their recruiting role	Patient care
		Appropriateness of participation
		Equipoise
	Research culture	Participation in other studies
		Communication
		Research team

At interview, patients focused on their experience of being injured and recovering from their fracture with little importance placed upon the trial. Patients’ and consultees’ experiences of the trial are reflected along with staff experiences within the theme ‘navigating research with patients after orthopaedic trauma’, while the themes ‘knowing it’s the right decision’ and ‘making it work’ were developed from the staff interview data. Quotes are included to illustrate our interpretation of participants’ accounts.

#### Navigating research with patients after orthopaedic trauma

Navigating involved engaging with patients and their families to i) support them in making sense of the study and ii) enable them to participate.

#### Making sense of the study

Patients eligible to participate in the study were described by staff to be typically frail, older patients. At interview, patients and consultees tended to recognise or recall the two interventions, knowing that one involved a piece of metal that attaches to the outside of the bone and one involved a piece of metal that goes through the middle of the bone. However, they could rarely describe the study in their own words. Several patients explained that around the time of their surgery, they were “that full of drugs I didn’t know what day it was” (patient 8) or not “in a fit state” (patient 5) to ask questions, reinforcing the difficulty in engaging with information at the time. Patients struggled to make sense of randomization and gave contradictory accounts of how their treatment was allocated despite it being explained to them again at interview. Some patients and consultees demonstrated therapeutic misconception, believing that they or their relative would receive the best treatment for them. This occurs “When a research subject fails to appreciate the distinction between the imperatives of clinical research and of ordinary treatment, and therefore inaccurately attributes therapeutic intent to research procedures” [17]. Others seemed confused about the alternative to trial participation, which they understood to mean no surgery rather than the surgeon choosing a method of fixation.

*They had to decide because they know what they are doing and I don’t.*

*Patient 5*

*I don’t mind, I don’t know what else they would have done with it. I know they didn’t put a plaster on my leg or anything like that.*

*Patient 9*

Staff felt that this group of patients were unlikely to comprehend and recall all elements of the study given their frail and at times confused state. Staff described knowing that both treatments are routinely used and that their surgeon was happy for them to participate as important to patients. They understood that the type of metalwork used to fix their fracture was unlikely to be a priority for patients who may not be able to appreciate the difference between the two interventions.

*Remember we are talking about the specific cohort of patients…when you have surgery, the drugs, the pain killers, a bit of fear, uncertainty all of these might be more important than whether you had a plate or a nail you know.*

*Surgeon 16*

*Some people are very trusting of the surgeons and don’t really care and don’t really understand the difference between a plate and a nail and as long as their problem gets fixed and something is being done they’re happy with either one or the other. It would be different if it was like you’re either getting a cast or an operation, that’s the difference but if they have an operation anyway …* RA 1

#### Enabling participation

At interview, patients and consultees expressed altruistic motivations for participation, wanting to help future patients or science. While they did not always understand and recall the study, they knew that they were helping in some way. Staff appreciated that patients wanted to help others and endeavoured to give them the opportunity to participate.

*I would love to, love to help and you know if there is anything I can do for, you know, research and things it helps doesn’t it, it helps other people.*

Patient 1

*I think that they know they’re helping, some of them have a bit more insight into some of the surgical studies maybe, but lots of them put their faith in the medical team that they know what’s best, “I don’t mind as long as I’m helping” they say, “I don’t mind as long as I’m fixed”.*

RA 5

RAs sought cues from patients to determine whether they could retain and weigh up information about the study in order to make an informed decision about participation. This included checking that patients could recall the study, spending time chatting with them and paying attention to the questions they asked.

*If they’re already in a study or it’s an observational study what we do when we’re not time pressured we actually go to see them one day, give them the information sheet, have a chat about the study. We go back the next day and if they’ve retained any of that conversation from yesterday or they remember who we are or they say they’ve read that sheet we gave them yesterday then that's our little method of assessing their capacity. We find that’s really useful for the borderline patients.*

*RA 12*

The majority of staff found involving relatives in the discussion about the trial enabled patients to be supported in their decision-making. They found this was particularly helpful for older patients who were often concerned about signing or agreeing to something without the involvement of their relative (typically their adult child).

*Sometimes you find that people can be quite nervous because… they’re told by their children that if somebody rings you up over the phone, don’t agree to do anything, don’t sign up for any contracts, don’t do anything… and all of a sudden you’re saying sign this and you can tell that they’re a bit guarded because it’s not something that they would normally do. Sometimes you can find it easier if you approach them to try and time it around their visiting hours and so they’ve got a relative with them and so you can kind of talk to them as a family and a lot more patients feel more comfortable with that.*

*RA 7*

A minority of staff, in contrast, found that it was easier to consent patients to studies without involving relatives. In their experience, involving relatives led to more patients declining. They found that relatives could be protective and felt that a research study may be too much for them after enduring trauma. Additionally, some staff found that relatives could also struggle to understand trials themselves.

*Through family, I have experienced more people declining generally for the fact that they’ve got a relative that’s undergoing something that’s really quite traumatic for the family and they don’t feel that they would want to put them through something else that is going to mean follow up or something else like that.*

*RA 4*

Patients who were entered into the study under nominated consultee agreement due to a lack of capacity and available personal consultee prior to surgery were approached to continue in the study when they regained capacity. RAs described feeling nervous, uncomfortable and guilty about approaching patients after surgery for consent to continue in the study. However, they found that the majority of patients accepted this procedure and did not mind having been already included.

*At first, we were quite sort of worried approaching people saying you’ve been randomised but actually… I don’t think we’ve had anyone that’s been miffed or upset.* *RA 11*

#### Knowing it is the right decision

Knowing that it is the right decision involved a process of interpreting the eligibility criteria, consideration of surgical preferences, skill and beliefs about equipoise with the intent of providing the best care for patients.

#### Interpreting the eligibility criteria

In this study, gaining confirmation from surgeons that patients were eligible for inclusion was challenging. Surgeons’ interpretation of the eligibility criteria was shaped by their beliefs about the suitability of both interventions for certain fractures. Patients with complex fractures were considered eligible by some surgeons but borderline by others.

*This study is focusing on a very narrow range of patients so they are very infrequent patients. Not all patients are suitable for both so patient selection is very difficult, sometimes they are borderline.*

*Surgeon 18*

*There are certain patients that would be eligible based on the criteria but whom people are saying no but this obviously needs a plate or no but this obviously needs a nail you would never do the other thing for this fracture. Now I appreciate that this may not be across sites but certainty within this site my perception is that patients are screened eligible but aren’t included because people are going that just shouldn’t have either nail or plate?*

*Surgeon 8*

#### Surgeon preferences

Surgeons’ own surgical preferences influenced their willingness to take part in this study. At interview, staff suggested that for simple distal femoral fractures, surgeons were typically in equipoise, were confident they could perform both procedures and were willing to randomise patients. However, staff reported that for complex fractures, surgeons were less willing to randomise patients. They explained that some surgeons believed that one method of fixation was more appropriate than the other for these fractures or that only one method of fixation was appropriate and “you would never do the other thing for this fracture” (Surgeon 8). Staff believed other surgeons either considered complex fractures too difficult to fix with a nail or had little, if any, experience of using nails.

*To give you an example the other day a patient came in with a periprosthetic knee and people felt unhappy to put a nail in… because they had never done it before.*

*Surgeon 20*

Surgeons emphasised that these fractures are uncommon and therefore most surgeons would have little experience fixing them. Furthermore, two surgeons explained that unlike many other operations, this operation is not performed from implicit memory, which could make it more difficult for surgeons to use their least preferred technique. Implicit memory uses previous experience to perform an activity without thinking about it.

*Distal femoral fractures are not common and so there are only about 10 percent of fractures of the femur overall and so no-one is doing lots unless you put your hand up to do them.*

*Surgeon 13*

*It’s not like a routine thing like an ankle fracture that you don’t really think about, it’s not like a brain stem reflex.*

*Surgeon 10*

Some staff described surgeons making decisions about treatment and trial participation as a group. In some centres, consultants were described as holding a collective preference for one of the two interventions or a consensus as to which patients they were prepared to include in the trial. Collective preferences for one of the two interventions could hinder recruitment, as it may be difficult to make the decision to randomise patients without the support of the group.

*…And someone else would pipe up well they’re eligible for TrAFFix and there’s a collective sigh of let’s hope it’s a plate then and so they have their preferences.*

*RA 5*

*For me I think that the real issue is that a consensus opinion falls before (TrAFFix) is considered, so everyone says this is what I think we should do so when you say so would you be happy to be randomised the consensus is no.*

*Surgeon 8*

#### Community equipoise

Of the surgeons interviewed, four expressed a personal preference for one of the two interventions but were still willing to randomise patients. This suggests that some surgeons accepted community equipoise (where uncertainty about the best treatment is held across the expert surgical community) and were able to proceed with their less preferred intervention. Furthermore, two surgeons emphasised that as individual equipoise can be difficult to achieve in surgical trials, it is important to accept community equipoise, which can give surgeons a reason to randomise patients.

*I don’t think when it comes to individual patients then surgeons can have that (equipoise), they always have an opinion about what they prefer to do because that’s what we’re trained to do. But I think the big real change for me over the… years really working in trauma research is that people have embraced this idea that as a community it’s okay for me to randomise my patient because as a group we don’t know. So the fact that I might have a preference individually at that particular moment for that particular patient with that particular fracture it’s still okay to randomise because as a community we don’t know.* *Surgeon 23*

#### Making it work

Making it work involved juggling a range of activities, balancing recruitment with concerns about study burden and developing a research culture within the department.

#### Juggling activities

RAs endeavoured to minimise the impact of research on clinical staff by juggling an array of activities and ensuring they fit in with clinical staff and their activities. They avoided taking too much of consultants’ time and tried to help where they could, for example by taking patients to the toilet or asking for pain relief or other medications for patients.

*You’ve got to try and make sure you’re not taking up their time because if the consultants have been delayed because of research and things their clinics are running over, the staff are having to stop later and it impacts (on) everybody and so I’m trying to make as little impact in the clinics as much as possible and on the ward areas. If you try and help for example with taking people to the toilet, you might go and see a patient and they say they want the bed pan and so you end up helping.*

*RA 16*

#### Balancing their own beliefs with their recruiting role

RAs endeavoured to balance their role of recruiting to research with their own beliefs about patient care. Some RAs described feeling caught between trial managers who wanted numbers and patients who needed care and time to reach a decision. They felt showing reciprocity and care to patients who had been approached about research during a time of vulnerability was important.

*Yes if I am with patients, I am with patients and that’s it because I want to have something with them and they deserve my time, I have to be there for them and to answer all their questions no matter how long it takes.*

*RA 1*

They also considered the appropriateness of research participation for patients with terminal diagnoses or multiple injuries.

*I’m a nurse and so obviously it’s a big consideration of mine if it’s not appropriate, if patients have had a terminal diagnosis then it’s very tricky when some of our studies follow the patients up for four months or six months.*

*RA 12*

Additionally, for two RAs, being aware that their surgeons may not have equipoise was troubling. They explained they would feel uncomfortable approaching patients or their relatives about participation in a trial if they were aware that their surgeon might prefer one of the two treatments. While they trusted the surgeon’s judgement, they needed to know that they were not causing harm to patients by asking them to participate.

*Yes for a surgical thing I would be very uncomfortable to approach someone saying the doctor’s happy to do either knowing that (the) surgeon had said I wouldn’t want to do that, I would rather do that. I wouldn’t like that at all.*

*RA 11*

*No, I think we need to know as well that the surgeons themselves could and will do either quite happily to a high quality because from our own emotional state that’s someone’s mother, someone’s daughter, someone’s loved one. You really want to know that what we’re doing is not causing any harm.*

*RA 12*

#### Research culture

Developing a research culture, where clinical teams are engaged in research could facilitate the delivery of research studies. In some centres, this was a “work in progress” (RA 4) while in others, staff described research to be routine. Three features that may foster the development of a research culture with departments were raised.

First, staff suggested that surgeons working in hospitals that are participating in several studies might become more involved in research and might identify or recruit patients for their colleagues’ studies as well as their own.

*And it’s taken a couple of years, there’s been a bit of resistance but now the PIs are getting on board and it’s “I’ll recruit to your trial if you recruit to my trial” and it’s a snowball effect.*

*Surgeon 13*

Second, enhanced communication from research teams to clinical staff might lead to a better understanding of ongoing research and engagement in the research process.

*I think in the bigger department there’s a lot of scepticism, people can be quite—not obstructive exactly but maybe they feel the researchers don’t communicate very well to them and so there seems to be this issue that people feel the studies happen and they weren’t told about it.*

*RA 14*

Third, staff also emphasised the importance of strong research teams to facilitate the development of a research culture and maximise recruitment to studies. Research teams endeavoured to promote studies, prompt recruitment and do the groundwork for surgeons.

*There’s a sort of assumption that we do all the groundwork for the surgeons, we highlight all the patients, we go prepared to the morning meetings so that we know what to expect and again it’s having that knowledge behind you that gives you the confidence to do that.* *RA 5*

## Discussion

The findings demonstrate how qualitative research makes a valuable contribution to evidence that highlights the challenges of conducting RCTs of surgical interventions. The central process of facilitating trial recruitment is conveyed through three themes, navigating research with patients after orthopaedic trauma, knowing it’s the right decision and making it work.

Navigating research with patients after orthopaedic trauma highlights the challenges of making sense of information whilst suffering the impact of injury and the importance of enabling participation through involving family and friends. At interview, participants’ recall of the study was poor and some misunderstood randomisation believing their surgeon chose the intervention they received. These findings are similar to that of Huxely et al. [12], who also found that while participants were willing to participate in an orthopaedic trauma trial, nearly all experienced some lack of recall. Our findings suggest that patients value the opportunity to participate in research with altruistic motivations for participation. Using the term ‘navigating’, previous research has described the way in which nurses signpost patients through healthcare systems [18, 19]. We found that RAs undertook this navigating role to support patients who were generally older and frail through the consent process and throughout research participation. Involving relatives in the discussion about the study was identified as important for this group of patients. This enabled patients who wanted to participate to be supported in doing so.

In knowing it’s the right decision, interviews with staff revealed a lack of individual surgeon equipoise, which many staff felt hindered recruitment to this trial. This is consistent with several previous studies which have highlighted the absence of clinicians’ individual equipoise [7, 20–22]. Lack of equipoise can undermine clinicians’ ability and willingness to recruit patients to trials and may influence the application of the eligibility criteria, which, similar to Ziebland et al. [23] and Hamilton et al. [22], we found differed between centres. Four surgeons interviewed in our study described willingness to randomise patients despite a personal preference for one of the two interventions, suggesting acceptance of community equipoise. The requirement of individual equipoise is debated. Community equipoise, characterised by a lack of satisfactory evidence and consensus among experts, could provide a justification for randomisation in the absence of individual equipoise [24]. Acceptance of community equipoise may therefore facilitate recruitment to surgical trials. For some surgeons, however, their unwillingness to randomise patients stemmed from a lack of experience of fixing distal femoral fractures. As these fractures are uncommon, some surgeons had little, if any, experience of using their least preferred intervention to fix them. This finding highlights the challenge of conducting surgical trials for uncommon conditions.

In making it work, the importance of juggling activities to fit in with clinical priorities, processing beliefs and values to integrate research into everyday practice and developing a research culture were key elements of facilitating trial recruitment. Our findings emphasise the importance of developing a strong research culture within the clinical team where clinical staff are engaged and informed about research. The presence of RAs embedded in the trauma service that can promote the studies and ensure they fit into everyday clinical practice could also help the development of a research culture. RAs can have a pivotal role in the success of a research team. By developing a strong relationship with clinical teams, they can act as a point of contact for the study, ensure that clinical staff feel informed and are engaged in ongoing research within the department and can prompt recruitment by highlighting potentially eligible patients.

### Strengths and limitations

Our sample of interviewees included patients, personal consultees, surgeons and RAs to capture the breadth of experience and allow corroboration of ideas. The valuable contribution of qualitative research through articulating the lived experience of participants is demonstrated through the three themes within facilitating trial recruitment. Staff and PPI representatives have indicated resonance with the findings. A greater degree of PPI throughout the study may have enhanced the presentation of these findings. Creative ways of involving patients are required to enable involvement of older, frail patients and those without capacity in the research process. No patients who declined to participate in TrAFFix agreed to be approached about the interview study. The experience of these patients may have provided a useful contribution to our understanding of what it is like to be approached about research participants after sustaining a fracture. Furthermore, inclusion of a wider range of staff such as physiotherapists and other multidisciplinary clinical staff may have allowed us to better understand how trials such as this will fit into clinical practice. Interviews with patients were conducted up to five months post-surgery. Although efforts were made to interview patients at earlier time points, this proved difficult and may have influenced their recall. Interviewing patients earlier may have afforded a greater understanding of patients’ experience of being approached to participate in a study and of the consent process.

Although this research was conducted within a trauma setting, these findings may be transferable to trials in other contexts. A detailed description of the participants, the TrAFFix study and our methods are provided to enable transferability of findings.

## Conclusions

Our findings raise three practical strategies for researchers involved in surgical trials within an emergency setting as well as areas for further research.

First, we found that our sample of older patients struggled to retain information about trial participation after orthopaedic trauma. Despite this, patients valued the opportunity to help others and contribute to science. Our findings suggest that involving relatives in the discussion about the study supported patients in their decision-making and participation in the study. Alternatively, taking a more proportionate approach to informed consent for research may help, as suggested by the NHS Health Research Authority (HRA) [25]. HRA guidance recommend potential participants to be provided with short, clear, relevant information that is proportionate to the risks and complexity of the research with further information available for those who wish to know more.

Second, our findings highlight the importance of understanding surgeons’ unwillingness to recruit patients to a trial. In this study, surgeons’ unwillingness stemmed from both a lack of equipoise and lack of experience using both surgical interventions. Developmental work to ascertain the views of stakeholders such as surgeons prior to undertaking a surgical trial could be valuable. This would allow research teams to understand the context in which the trial will be conducted, identify the likely barriers and to design the most appropriate approach such as expertise-based randomised controlled trials, where clinicians only perform the intervention they are expert in [26].

Third, our findings suggest that building a research culture within the clinical team where research is part of everyday practice may facilitate recruitment. In this study, we found that RAs were considered to have a crucial role, which included promoting studies and doing the groundwork for surgeons.

Two areas for further research were highlighted. First, further research should seek to identify additional strategies to support older patients to make sense and recall the information they are given to enable them to participate in research. Second, further research is needed to understand surgeons’ views of community equipoise. Our findings showed that for some surgeons the presence of community equipoise enabled them to include their patients in the study despite a lack of individual equipoise. Exploring whether reinforcing the presence of community equipoise among surgeons may facilitate recruitment to difficult trials where individual equipoise is absent may be valuable.

## Data Availability

We do not have consent for data storage in a repository. For further information, contact the corresponding author.
